# Circadian regulation in aging: Implications for spaceflight and life on earth

**DOI:** 10.1111/acel.13935

**Published:** 2023-07-26

**Authors:** Deeksha Malhan, Britt Schoenrock, Müge Yalçin, Dieter Blottner, Angela Relόgio

**Affiliations:** ^1^ Institute for Systems Medicine and Faculty of Human Medicine MSH Medical School Hamburg Hamburg Germany; ^2^ Institute of Integrative Neuroanatomy Charité‐Universitätsmedizin Berlin, Corporate Member of Freie Universität Berlin, Humboldt‐Universität zu Berlin, and Berlin Institute of Health Berlin Germany; ^3^ Institute for Theoretical Biology (ITB) Charité—Universitätsmedizin Berlin, Corporate Member of Freie Universität Berlin, Humboldt‐Universität zu Berlin, and Berlin Institute of Health Berlin Germany; ^4^ Molecular Cancer Research Center (MKFZ), Medical Department of Hematology, Oncology, and Tumour Immunology, Charité—Universitätsmedizin Berlin, Corporate Member of Freie Universität Berlin, Humboldt‐Universität zu Berlin, and Berlin Institute of Health Berlin Germany; ^5^ Neuromuscular System and Neuromuscular Signaling Berlin Center of Space Medicine & Extreme Environments Berlin Germany

**Keywords:** aging, biological aging, circadian clock, circadian rhythms, space exploration, spaceflight

## Abstract

Alterations in the circadian system are characteristic of aging on Earth. With the decline in physiological processes due to aging, several health concerns including vision loss, cardiovascular disorders, cognitive impairments, and muscle mass loss arise in elderly populations. Similar health risks are reported as “red flag” risks among astronauts during and after a long‐term Space exploration journey. However, little is known about the common molecular alterations underlying terrestrial aging and space‐related aging in astronauts, and controversial conclusions have been recently reported. In light of the regulatory role of the circadian clock in the maintenance of human health, we review here the overlapping role of the circadian clock both on aging on Earth and spaceflight with a focus on the four most affected systems: visual, cardiovascular, central nervous, and musculoskeletal systems. In this review, we briefly introduce the regulatory role of the circadian clock in specific cellular processes followed by alterations in those processes due to aging. We next summarize the known molecular alterations associated with spaceflight, highlighting involved clock‐regulated genes in space flown *Drosophila*, nematodes, small mammals, and astronauts. Finally, we discuss common genes that are altered in terms of their expression due to aging on Earth and spaceflight. Altogether, the data elaborated in this review strengthen our hypothesis regarding the timely need to include circadian dysregulation as an emerging hallmark of aging on Earth and beyond.

## Graphical abstract

Circadian dysregulation as an emerging hallmark of aging on Earth and during space travel.

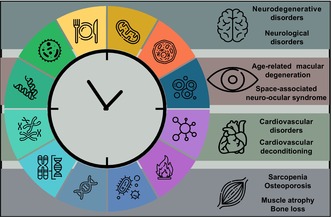

**Abbreviations**
AD: alzheimer's disease
AMD: age‐related macular degeneration
CCG: clock‐controlled genes
CNS: central nervous system
CSA: canadian space agency
ESA: european space agency
HRP: human research program
HUVEC: human umbilical vein endothelial cells
ISS: international space station
JAXA: japan aerospace exploration agency
NASA: national aeronautics and space administration
PD: parkinson's disease
ROS: reactive oxygen species
RPE: retinal pigment epithelium
SANS: space‐associated neuro‐ocular syndrome
SCN: suprachiasmatic nucleus
TTFL: transcriptional‐translational feedback loop

## 1 INTRODUCTION

Aging is a complex process that involves a time‐related decline in several molecular and cellular processes, and ultimately also in physiological functions necessary for survival and fertility in an organism. Aging phenotype results from alterations in telomere length, epigenetic regulation, and mitochondrial function, along with dysregulations in nine other processes (proteostasis, macroautophagy, cellular senescence, stem cell exhaustion, intercellular communication, chronic inflammation, dysbiosis, genomic instability, and nutrient‐sensing), which are altogether referred to as the hallmarks of aging (López‐Otín et al., 2023). Interestingly, recent studies associate aging to spaceflight with contradictory conclusions (Biolo et al., 2003; Cannavo et al., 2022; Garrett‐Bakelman et al., 2019; Honda et al., 2012; Ma et al., 2015; Malhan et al., 2023; Nwanaji‐Enwerem et al., 2020; Otsuka et al., 2019; 2022; Wang, 1999), while some of the studies report an anti‐aging impact of spaceflight, others observed progressive aging due to spaceflight. Whether the anti‐aging impact of spaceflight correlates with the “twin paradox” (Gron, 2006) based on the theory of relativity coined by Albert Einstein, which proposes that an individual in Space ages slower compared to his/her biological twin on Earth, remains to be shown. Increased focus on Space exploration and the required lengthening of Space missions for Deep Space Planetary exploration, increases the need for a better understanding of Space mission‐associated alterations on aging‐related pathways with impact on the health status of humans in Space.

The HRP (Human Research Program) carried out by the NASA (National Aeronautics and Space Administration) has recently reported five major human spaceflight‐related hazards for long Space missions with a focus on future missions to Mars. These include exposure to Space radiation, altered gravity fields, isolation and confinement, closed environment, and distance from Earth (Patel et al., 2020). These hazards are linked with several risks to human health ranked according to their potential impact on the crew's health (red: higher risk, yellow: medium risk, and green: controllable risk). The HRP‐defined “red flag” risks include SANS (Space‐Associated Neuro‐Ocular Syndrome), cardiovascular disease, behavioral and cognitive impairment, circadian dysregulation, and muscle wasting due to exposure to microgravity (HRR–Evidence (nasa.gov)).These “red flags” are categorized into the four most affected systems as defined by International Space Agencies like NASA, ESA (European Space Agency), or JAXA (Japan Aerospace Exploration Agency): visual, cardiovascular, central nervous, and musculoskeletal systems. Similar pathological alterations also occur as a consequence of aging on Earth. As the crew members are at a greater risk of experiencing these health stressors, it is important to understand their specific and combined impact on human performance and health in Space.

One major alteration to which astronauts are subjected during Space missions and which is related to the stressors described above is the disruption of their endogenous biological rhythm generated by the circadian clock (Guo et al., 2014). The circadian clock plays a regulatory role in an optimal adaptation of behavior and physiology to the 24 h Earth's rotation (Olejniczak et al., 2023), thereby generating ~24 h (circadian) rhythms, at the cellular level, in the human body. Cellular clocks are, under normal conditions synchronized with the main clock located in the diencephalic brain (epithalamus and hypothalamus), which in turn is entrained to the external environment (light, temperature), and thus contributes to the regulation of physiological features, for example, the sleep/wake cycle (Yadlapalli et al., 2018). On low Earth orbit, astronauts experience a sunrise or sunset every 45 min, resulting in circadian clock dysregulation with a significant impact on circadian‐regulated biological pathways and subsequently on the timing of cellular processes, physiology, and behavior. Previous evidence points to disruptions of the circadian clock being associated to aging and aging hallmarks on Earth (Benitah & Welz, 2020), and increased incidence of age‐related diseases such as macular degeneration (Blasiak et al., 2022), cancer (López‐Otín et al., 2023), and neurodegenerative disorders (Shen et al., 2023).

**FIGURE 1:**
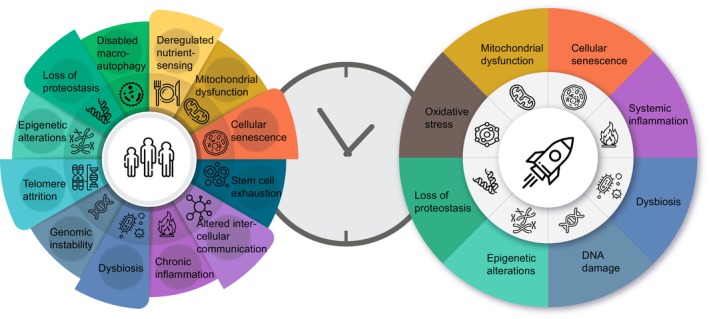
Schematic representation of altered biological processes due to aging on Earth (left side) and Space exploration journey (right side).

Common features of aging on Earth and microgravity environment‐induced alterations have been reviewed by Biolo et al. and include muscle mass loss, mitochondrial dysfunction (Figure 1), and systemic inflammatory response (Biolo et al., 2003). Nevertheless, additional supporting evidence is needed to determine if indeed long‐duration spaceflight affects human health in the long run, thus possibly affecting aging processes during future extended Space missions, as well as affecting the lifespan of astronauts after returning to Earth. This becomes more and more relevant the deeper we explore and even colonize other planets requiring long‐term exposure to different environments not just in terms of gravity, but also in terms of variable day length durations. The later may even more negatively affect astronauts through circadian disruption, and subsequent requirements for readaptation to new circadian, possibly ultradian (shorter than 24 h) and infradian (longer than 24 h) cycles, for example, in the next generation of extraterrestrial human life.

In this review, we discuss the current knowledge on the circadian clock in both aging on Earth and aging‐related alterations during Space travel. Following an overview on the relevance of circadian rhythms and their role in several cellular processes in Section 2.1. We then describe the impairments of the core‐clock system and clock‐controlled genes in four major age‐related health risks: vision decline, cardiovascular diseases, CNS (central nervous system) damage, and musculoskeletal deterioration associated with aging (Section 2.2), as well with spaceflight (Section 3). Finally, we provide a brief perspective on the future needs in terms of Space exploration to allow for a more mechanistic understanding regarding the aging process and the putative, still largely unstudied, role of circadian (dys)regulation in contribution for its control and overall health management of human life in the future.

## 2 A LINK BETWEEN THE CIRCADIAN CLOCK AND AGING

As the world's population continues to age, there has been a significant increase in the number of elderly individuals aged 60 years and older from 12% to 22% projected by 2050 (World Health Organization). Therefore, investigating the impact of aging on health and finding ways to slowdown the process and improving quality of life for the elderly population is becoming increasingly important. Given its major relevance for maintenance of health and cumulating evidence pointing toward the influence of the circadian system in the physiological alterations of aging‐related processes, we here propose to include circadian dysregulation as an emerging hallmark of aging, as an overdue hypothesis and rationale to better understand aging and longevity aspects of life on Earth and in Space now and in near future.

### 2.1 Circadian regulation of cellular processes

**FIGURE 2:**
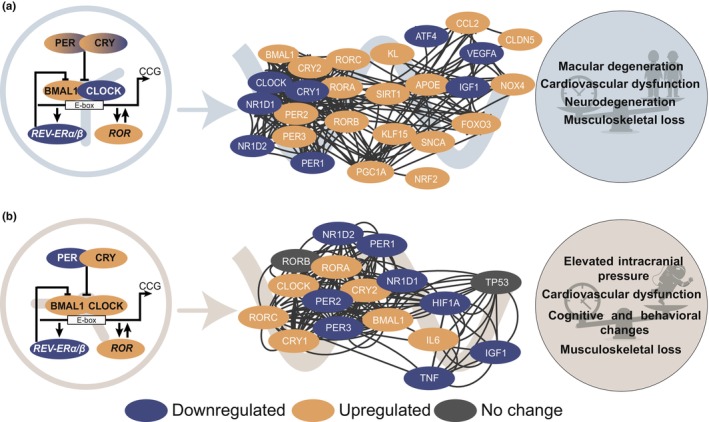
Alterations in the core‐clock machinery induce expression variations in clock‐regulated genes associated with aging on Earth and increased risk of health problems due to spaceflight. Circadian transcriptional–translational feedback loops generate robust rhythms under healthy conditions, which may be dysregulated due to (a) aging on Earth, and (b) extreme environmental conditions such as spaceflight, resulting in increasing health risks if not developing pathophysiologic mechanisms possibly ending up in severe disease states.

The body's daily rhythm is described as “circadian”, derived from Latin words circa (around) and *diem* (day). These ~24 h rhythms exist in numerous living forms on Earth, from single‐celled (e.g., cyanobacteria, prokaryotes) to multicellular and nucleated organisms (eukaryotes) including plants, insects, and humans (Neves et al., 2022). The emergence of whole genome transcriptomics in the field of chronobiology helped in exploring temporal gene expression among cells and tissues. In mammals, more than 50% of the transcriptome is shown to be rhythmically expressed with ~24 h oscillations in at least one organ (Cheng et al., 2019; Zhang et al., 2014). In addition to circadian rhythms, evolutionary conserved genes among different species are reported to exhibit rhythms with a shorter period duration (8 h or 12 h) known as ultradian (Castellana et al., 2018). Moreover, different bird and mammalian species exhibited rhythms in body temperature with a shorter period duration (20 min to a few hours), known as episodic ultradian events (Goh et al., 2019). Besides ultradian rhythms, in different species, several physiological parameters including menstruation cycle and memory function exhibit rhythms with a longer period duration (about 4 days) known as infradian (Diatroptov, 2021; Hartsock et al., 2022; Laje et al., 2018). Circadian rhythms are generated at the molecular level in mammals, by a cell‐autonomous TTFL (transcriptional‐translational feedback loop) that consists of core‐clock genes including *CLOCK*, *BMAL1* (also known as *ARNTL*), *PER1/2/3*, *CRY1/2*, *RORA/B/C*, and *NR1D1/2*. This interconnected network drives the circadian expression of output target genes known as CCGs (clock‐controlled genes; Figure 2; Rijo‐Ferreira & Takahashi, 2019).

The circadian clock coordinates the timing of several physiological processes including retinal function (Bhoi et al., 2022), cardiac metabolism (Luo et al., 2022; Schroder & Delisle, 2022), neural activity (Reiter et al., 2023), and myogenesis (Zhang et al., 2020). Among different biological processes, the circadian clock regulates the expression and activity of mitochondrial metabolic and antioxidant enzymes (Mezhnina et al., 2022; Scrima et al., 2020). To ensure the daily coordination of these processes via environmental cues, diurnal light variations are conveyed by the retina to a central pacemaker, located in the SCN (suprachiasmatic nucleus) in the diencephalic brain hypothalamus (Ono, 2022). Light is a central zeitgeber (“time giver”) for entraining the circadian clock to its external environment (Lorber et al., 2022). Besides light, physical exercise (Kim et al., 2023), and food (Lewis et al., 2020) for example, may serve as zeitgebers. Physiological and organ functions are thus, via the clock, also entrained to the normal daily cycles of life on Earth.

#### 2.1.1 Visual system and circadian regulation

**FIGURE 3:**
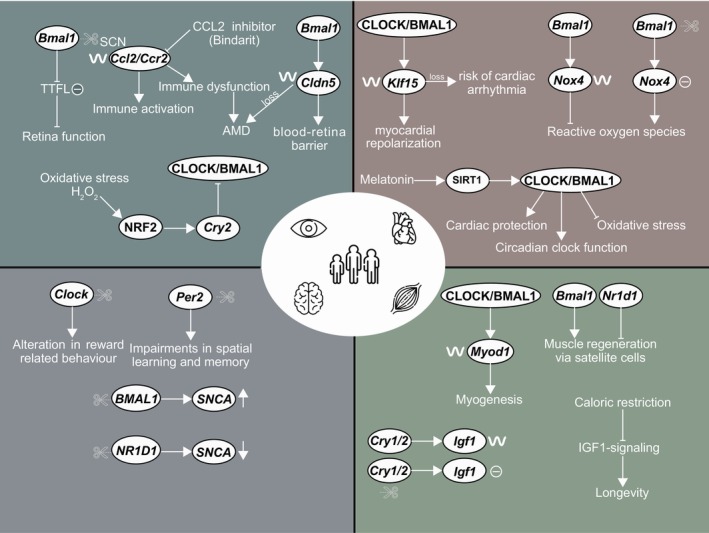
Graphic representation of the pathophysiological roles of core‐clock genes in age‐associated diseases. Core‐clock genes play a crucial role in the whole‐body homeostasis and their loss/gain influence visual, cardiovascular, central nervous system, and musculoskeletal system as depicted in the regulatory networks.

The circadian clock plays a major role in the maintenance of vision and its disruption can result in retinal degeneration (Jauregui‐Lozano et al., 2022). In retina function, *Bmal1* plays an important role in the maintenance of circadian rhythms, and the null mutation of *Bmal1* in mice affected clock‐regulated processes by altering the TTFL (Figure 3; Storch et al., 2007). Another core‐clock gene, *Per1* is rhythmically expressed in cells of the inner retina of mice, and is essential for the maintenance of retinal health (Witkovsky et al., 2003). Besides retina neurons, the so‐called “Müller” cells (retinal glial cells) were the first mammalian cell type that were reported, via bioluminescence signal detection, to have circadian expression in isolation from other retinal cells (Xu et al., 2016). A circadian system‐related hormone, melatonin, synchronizes circadian rhythms and related physiological functions via G‐protein‐coupled receptors. Melatonin is synthetized in the central pituitary gland of the epithalamus (diencephalon) and stimulated by the SCN via retinal ganglionic axon terminals as a part of a parallel fibre tract, in addition to the main optic nerve and tract fibres that target to the main vision areas in the occipital lobe of the brain. Melatonin synthesis in the pituitary gland (epiphysis) is high during night and usually lowered by normal daylight or other artificial light sources (eye perception) (Zhao et al., 2019). The circadian regulation of melatonin synthesis is mediated by the retino‐hypothalamic pathway and the specialized melanopsin‐containing neurons directly project the light input to the SCN, suppressing the production of melatonin (Lundmark et al., 2006). In mammals, light is perceived by retinal ganglion cells (ipRGCs), in addition to rods and cones photoreceptors. ipRGCs play an important role in circadian entrainment, and they respond to light through the suppression of nocturnal melatonin (Mure, 2021). Melatonin is also an effective antioxidant, and several ocular tissues including the corneal epithelium, dopaminergic amacrine neurons contain melatonin receptors, and thus melatonin can protect ocular tissues against oxidative stress (Lundmark et al., 2006). Besides pineal glands, melatonin can be synthesized in various organelles, for example, mitochondria, which is a major source of ROS (reactive oxygen species), and therefore plays a role in redox homeostasis crucial for the maintenance of human health (Melhuish Beaupre et al., 2021). However, melatonin levels decline with aging, thereby weakening its protective effect against oxidative stress in these tissues (Bondy, 2023).

#### 2.1.2 Cardiovascular system and circadian regulation

In the cardiovascular system, the circadian clock regulates several key mechanisms such as blood pressure (Costello & Gumz, 2021), heart rate (El Jamal et al., 2023), and cardiac contractility (Latimer & Young, 2022). *BMAL1* expression is crucial for mitochondrial activities and cardiac function, and its deletion in human embryonic stem cells reduced BNIP3 protein expression and led to dilated cardiomyopathy (Li et al., 2020). Another clock gene *dCry* plays an important role in heart morphogenesis and function in *Drosophila* (Alex et al., 2015). Metabolic genes regulated by PPARα: *Pdk4* and *Ucp3* showed circadian expression in the rat heart and their circadian expression was regulated by NR1D1, essential for myocardial metabolism (Durgan et al., 2005). Several other genes associated with electrophysiological activity including *Glut1*/*4* (Young et al., 2001), and potassium ion channels (Kv1.5 and Kv4.2) showed circadian expression in rat cardiomyocytes (Yamashita et al., 2003).

#### 2.1.3 Neuronal plasticity and circadian regulation

Previous studies have shown that the core‐clock genes *Clock*, *Bmal1*, and *Per1/2* are involved in regulating behavioral outputs within the CNS (Snider et al., 2018; Vadnie & McClung, 2017). For example, mice with a *Clock* deletion exhibited altered reward‐related behavior and impulsivity, pointing to other mood or behavioral changes such as hyperactivity (Spencer et al., 2012), while *Per2* knockout mice displayed impairments in spatial learning and memory, and elevated expression of *Drd1* gene (Kim et al., 2022). *Bmal1* knockout mice showed altered circadian patterns of locomotor activity and behavioral changes such as impairment to habitual adaptation (Kondratova et al., 2010). Knockout of the core‐clock gene *Bmal1* or *Clock*/*Npas2* double knockout in mice can lead to impaired neuronal plasticity in the brain hippocampus and cerebral cortex, regions critical for learning and memory (Musiek et al., 2013). The interplay between the *Bmal1* and *Per2* and CCGs such as *GSK3β*, a regulator of glucose metabolism and inflammation, was also implicated in regulating neuronal excitability and synaptic plasticity (Besing et al., 2017). Inhibition of GSK3 shortened the period of hippocampal molecular clock evaluated via *PER2* reporter activity, whereas its activation resulted in disruption in BMAL1 rhythms suggesting a role for the *GSK3* activity on the molecular clock (Besing et al., 2017). These findings suggest that disruption of core‐clock genes and CCGs can have a significant impact on behavioural and neural outputs of the CNS.

#### 2.1.4 Musculoskeletal system and circadian regulation

In the musculoskeletal system, the circadian clock regulates several functions such as maintenance of skeletal muscle phenotype (McCarthy et al., 2007) and bone metabolism (Qin et al., 2023). Previous studies using mice showed that BMAL1 and CLOCK directly regulate the circadian expression of *MyoD*, which is a master regulator of myogenesis (Andrews et al., 2010). Moreover, *Bmal1* plays a crucial role in muscle regeneration via satellite cell (i.e., typical muscle stem cell type) expansion, and loss of *Bmal1* in mice displayed reduced satellite cell growth ex vivo, and lower expression of satellite cell marker Pax7 (Chatterjee et al., 2015). NR1D1, a suppressor of *Bmal1* was later reported as a novel inhibitory regulator of muscle regeneration, and loss of *Nr1d1* in mice showed enhanced proliferation of satellite cells (Chatterjee et al., 2019). In addition to skeletal muscle, the circadian clock plays a crucial role in bone metabolism (Hirai, 2017). Bone homeostasis is tightly orchestrated by the activity of osteoblasts (bone formation and mineral accretion) and osteoclasts (bone resorption) (Maciel et al., 2023). The core‐clock genes *Per2* and *Cry2* are shown to affect bone homeostasis in mice via influencing osteoblast and osteoclast activity, respectively (Maronde et al., 2010). Furthermore, several reports showed that bone formation is prevalent during the day, while bone resorption is prevalent during the night, following circadian rhythms (reviewed by (Tian & Ming, 2022)). Loss of *Bmal1* displayed low bone mass phenotype in a mouse model (Samsa et al., 2016). Taken together, these results suggest that the core‐clock machinery plays an important role in regulating different components of the musculoskeletal system.

### 2.2 Aging and functional dysregulation of cellular processes

Twelve hallmarks of aging have been defined, which include cellular and organismal processes dysregulated in aging: mitochondrial dysfunction, genomic instability, dysbiosis, chronic inflammation, altered intercellular communication, stem cell exhaustion, cellular senescence, deregulated nutrient‐sensing, disabled macroautophagy, loss of proteostasis, telomere attrition, and epigenetic alterations (Figure 1; left panel) (Lopez‐Otin et al., 2023). The complexity of aging process relies on the mutual networking of these aging hallmarks. Several of those being similarly affected during spaceflight (Figure 2; Garrett‐Bakelman et al., 2019) including the visual system (Owsley, 2011), cardiovascular system (Paneni et al., 2017), CNS (Swenson et al., 2019), and musculoskeletal system (Roberts et al., 2016), as mentioned in the Introduction (Section 1). In addition, aging is associated with alterations in circadian rhythms, which in turn negatively impact health (Zhu et al., 2022). In the following section, we will focus on the four key systems listed above and introduce literature evidence concerning the dysregulation of circadian rhythms in the context of aging.

#### 2.2.1 Visual system and aging

Aging has a major impact on the deterioration of vision components. Aging‐related alterations in ocular tissues may lead to dry eye disease, macular degeneration, and even blindness (Guymer & Campbell, 2023). Among different known eye pathologies, AMD (age‐related macular degeneration), mainly characterized by degeneration in neuroepithelium of macular area, remains a leading cause of vision loss or blindness in different countries (Wong et al., 2014). Despite the increased knowledge on the molecular complexity underlying AMD, little is known about the role of the circadian clock in this pathogenesis. In a genome wide study with 88 AMD cases and 91 controls, *APOE* polymorphism in E2 allele showed a strong correlation with the increased risk of early AMD (Klaver et al., 1998). Another study showed that the loss of monocyte and macrophage chemoattraction‐associated genes *Ccl2* and *Ccr2* in mice lead to macrophage dysfunction (Table 1), thereby contributing to early AMD pathology (Ambati et al., 2003). Both *Ccl2* and *Ccr2* expression showed rhythmic profile in the SCN in mice and CCL2 inhibition in the SCN diminished the circadian activation of immunity (Duhart et al., 2016). In a case–control study, *ELOVL4* and *CFH* genes demonstrated a significant association with the status of late AMD (Conley et al., 2005). Another gene from the ELO family, *Elovl2* promoter region showed increased methylation with age in mice retina, suggesting a potential role of *Elovl2* as a regulator of molecular aging clock in retina (Chen et al., 2020). Moreover, *Elovl2* and *Elovl4* expression are core‐clock regulated as they depicted circadian expression in control rat retina, while their circadian expression was lost or diminished in a diabetic rat retina (Wang et al., 2014). Decline in the capacity of antioxidant molecules within RPE (retinal pigment epithelium), the epithelial cell layer within neurosensory area of retina, is a hallmark of AMD (Abokyi et al., 2020). In human RPE cells exposed to arsenite (oxidative stress), ATF4 (activating transcription factor; important for redox processes) lead to a direct increase in VEGF expression, a contributing factor to wet AMD (Roybal et al., 2005). Later, it was shown that the loss of *Pten* gene, which is important for the maintenance of RPE cell junction integrity, resulted in retinal degeneration in mice via increased oxidative stress (Kim et al., 2008). *Nrf2* acts as a master regulator of endogenous antioxidant protection, and a study by Zhao Z et al. reported that the loss of *Nrf2* in mice developed an age‐dependent degeneration of the RPE cells (Zhao et al., 2011). Another study showed that binding of NRF2 to *Cry2* gene, led to the inhibition of CLOCK/BMAL1‐mediated transcription in mice hepatocytes (Wible et al., 2018). Furthermore, loss of *Nrf2* in mice fibroblasts, hepatocytes, and liver resulted in circadian alterations, depicting a cross‐talk between *Nrf2* and the circadian clock. In a recent study, *Dapl1* deficiency in mice impaired the antioxidant capacity of RPE through inhibition of MITF and its targets (NRF2 and PGC1A) that are needed for antioxidant defence mechanisms (Ma et al., 2023). PGC1α, essential for energy metabolism, is known to regulate different components of the core‐clock network including *Bmal1* (Liu et al., 2007). Another study depicted that the knockout of *POLDIP2* in human RPE cell line contributes to higher risk of AMD through reduction in mitochondrial superoxidase levels and upregulation of *SOD2* gene, which is a mitochondrial superoxidase dismutase (Nguyen et al., 2023). Polymorphism in several other oxidative stress‐associated factors including, *HMOX1* and *HMOX2* (Synowiec et al., 2012) and alternative complement pathway‐associated factor *CFD* (Stanton et al., 2011) are associated with the occurrence and progression of different forms of AMD (dry and wet) among patients versus controls. Furthermore, BMAL1 in mice regulates the expression of *Cldn5*, which is required for the functional integrity of inner blood‐retina barrier, and loss of *Cldn5* is associated with dry AMD pathology (Hudson et al., 2019). In summary, the visual system is highly sensitive to the aging process and is a relevant bridging element between the circadian clock and aging. Several other genes associated with the risk of AMD are regulated by the circadian clock. On the other hand, important zeitgebers like light require specific cells and paths within the visual system, to allow for entrainment of the endogenous clock with the geophysical time, and the functioning of such cells decline with aging, with a negative impact on circadian regulated cellular and physiological processes.

**TABLE 1:** Association between core‐clock regulated genes and aging‐related diseases.

	Gene	Species	Mutation/loss/gain	Main findings	References
Visual system and aging	*Apoe*	Human	apoE alleles (E2 allele: increased risk of AMD E4 allele: decreased risk of AMD)	apoE polymorphism is significantly associated with the risk of AMD	Klaver et al. (1998)
	*Ccl2/Ccr2*	Mouse	Loss of *Ccl2* or *Ccr2*	Deficiency of *Ccl2* or *Ccr2* in mice leads to AMD	Ambati et al. (2003)
	*ELOVL4*	Human	Met299Val variant	*ELOVL4* polymorphism is significantly associated with the early onset of age‐related maculopathy	Conley et al. (2005)
	*CFH*	Human	Tyr402His variant	*CFH* polymorphism is significantly associated with the early onset of age‐related maculopathy	Conley et al. (2005)
	*Elovl2*	Mouse	*Elovl2* promoter region methylation and expression decline	*Elovl2* expression declines with aging affecting vision and *Elovl2* mutation is associated with risk of AMD	Chen et al. (2020)
	*ATF4*	Human RPE cells	ATF4 protein level	Increase in ATF4 protein level due to oxidative stress contribute to AMD	Roybal et al. (2005)
	*VEGFA*	Human RPE cells	*VEGFA* mRNA level	Increase in *VEGFA* via ATF4 contribute to AMD	Roybal et al. (2005)
	*Pten*	Mouse	Loss of *Pten*	Loss or inactivation of PTEN can result in AMD	Kim et al. (2008)
	*Nrf2*	Mouse	Loss of *Nrf2*	*Nrf2* ^*−/−*^ mice developed age‐dependent degeneration in RPE	Zhao et al., 2011)
	*Dapl1* MITF PGC1A	Mouse Human RPE cells	Loss of *Dapl1*	DAPL1 binds to MYC and result in upregulation of MITF and its targets (NRF2, PGC1A), which prevent against oxidative stress. Deficiency of *Dapl1* in mice impair the antioxidant capacity and lead to AMD	(Ma et al. (2023)
	*POLDIP2* *SOD2*	Human RPE cells	Loss of *POLDIP2*	Deletion of *POLDIP2* in RPE cells resulted in lower superoxide level via upregulation of *SOD2*. Loss of *POLDIP2* increases the risk of AMD	Nguyen et al. (2023)
	*HMOX1* *HMOX2*	Human	Polymorphism (G to C/ A to G transition)	*Hmox1* and *Hmox2* polymorphism is associated with dry AMD, progression of dry to wet AMD	(Synowiec et al., 2012)
	*CFD*	Human	Polymorphism and plasma level	Plasma CFD concentration was significantly higher in AMD patients vs. controls	(Stanton et al., 2011)
	*Cldn5*	Mouse	Suppression of *Cldn5* expression	Targeted suppression of Cldn5 lead to RPE atrophy	(Hudson et al., 2019)
Cardiovascular system and aging	*Foxo3*	Mouse	Loss of *Foxo3*	Targeted deletion of *Foxo3* enhanced calcineurin signaling and cardiac growth	(Ni et al., 2006)
	*KLF5*	Human Mouse	Overexpression of *KLF5*	*KLF5* expression regulated via FOXO1 is associated with cardiac dysfunction	(Kyriazis et al., 2021)
	*Nrf2*	Human Mouse	Increase in *Nrf2* mRNA level	*Nrf2* is linked with the acceleration of pathogenesis of atherosclerosis	(Kloska et al., 2019)
	*Nox4*	Human Mouse	Increase in *Nox4* mRNA level	Elevated expression of *Nox4* is associated with several cardiovascular diseases	(Chen et al., 2012)
	*Cx43*	Human Mouse	Change in *Cx43* expression	Cx43 expression and localization is altered with age and in age‐associated cardiovascular diseases	(Michela et al., 2015)
	*PGC1A*	Human Mouse	Downregulation of *PGC1A* at gene and protein level	PGC1A expression is altered in different mouse models of heart failure and in patients	(Oka et al., 2020)
	*VEGFA*	Human	Alterations in VEGFA level	Higher level of VEGFA is observed in patients with cardiovascular diseases	(Braile et al., 2020)

	*TSP1*	Mouse	Loss of *TSP1*	Deficiency of *TSP1* in mice led to early onset of heart hypertrophy	(Chistiakov et al., 2017)
	*KL*	Human	KL‐VS allele	Individual with homozygous KL‐VS variant at a greater risk of stroke	(Arking et al., 2005)
	*SIRT1*	Mouse	Overexpression of *Sirt1*	Higher level of *Sirt1* overexpression lead to cardiomyopathy	(Alcendor et al., 2007; Soni et al., 2021)
*Klf15*	Mouse	Loss or overexpression of *Klf15*	Loss of *Klf15* in mice increased the susceptibility to arrythmia	(Jeyaraj et al., 2012)
CNS aging‐related alterations	*PSEN1* *PSEN2*	Human	Mutation in *PSEN1* and *PSEN2*	Mutation in *PSEN1* and *PSEN2* is significantly associated with early onset AD	(Lanoiselee et al., 2017)
	*MAPT*	Human	MAPT H2 haplotype	H2 haplotype of *MAPT* is associated with the risk of late onset AD	(Allen et al., 2014)
	*APOE*	Human	APOE E4 allele	Elevated expression of APOE and polymorphism in E4 allele is associated with AD risk	(Bessi et al., 2020) (Hoyt & Obrietan, 2022)
	*SNCA*	Human	Change in SNCA expression	Elevated *SNCA* mRNA levels were observed in dopaminergic neurons from idiopathic PD brains vs. controls	(Gründemann et al., 2008)
	*PARK2*	Human	Mutation in *PARK2* gene	Mutation in *PARK2* gene is associated with autosomal recessive juvenile parkinsonism	(Lunati et al., 2018)
Musculoskeletal system and aging	*Atf4*	Mouse	Muscle specific deletion of *Atf4*	*Atf4* knock out mice exhibited less age‐related muscle atrophy than controls	(Miller et al., 2023)
	*ACTN3*	Human	*ACTN3* R577X polymorphism	Elderly women with *ACTN3* polymorphism showed a strong correlation of developing sarcopenia	(Romero‐Blanco et al., 2021)
	*NRF2*	Human	*NRF2* rs12594956 polymorphism	*NRF2* polymorphism showed a strong correlation with the risk of sarcopenia	(Urzi et al., 2020)
	*IGF1*	Human	*IGF1* rs35767 polymorphism	*IGF1* polymorphism in elderly population is associated with alterations in body composition	(Kostek et al., 2010; Pratt et al., 2019)
	*TNFA*	Human	*TNFA* rs361525 polymorphism	Males with *TNFA* polymorphism showed significant better physical performance level compared to their controls	(Pratt et al., 2019; Tiainen et al., 2012)
	*CNTF*	Human	*CTNF* polymorphism (rs948562, rs1800169, rs550942, rs4319530, rs1938596)	Women with *CNTF* polymorphism showed significant differences in muscle strength	Arking et al. (2006); Pratt et al. (2019)
	*UCP3*	Human	*UCP3* rs1800849 polymorphism	Elderly with *UCP3* polymorphism showed better muscle strength than their controls	Crocco et al., 2011; Pratt et al. (2019)
	*Dkk3*	Human Mouse	Expression change in *Dkk3*	Forced expression of *Dkk3* in young mice led to sarcopenia Elevated Dkk3 level in sarcopenia patients	Yin et al. (2018)
	*CUL1* *PTEN* *STAT1*	Human	Change in gene expression level	Higher expression of *CUL1*, *PTEN*, and *STAT1* was significantly associated with osteoporotic patients vs. controls	Deng et al. (2023)

#### 2.2.2 Cardiovascular system and aging

Cardiovascular diseases like coronary heart disease and atherosclerosis present a major burden on aging society worldwide (Liberale et al., 2022).

Tuzcu et al. carried out intravascular ultrasound on heart transplant recipients and reported higher prevalence of atherosclerosis among subjects over 50 years old (85%) when compared to subjects under 20 years old (17%) (Tuzcu et al., 2001). In recent years, several genes have been identified in connection with age‐associated cardiovascular diseases. Among the different key regulators found, a gene polymorphism in *FOXO3* is associated with longevity, and was shown to be important for the maintenance of cardiovascular homeostasis (Zhao & Liu, 2021). *Foxo3* was reported to be important to repress cardiac growth, its deletion in mice resulted in a hypertrophic phenotype compared to controls (Ni et al., 2006). Cardiac dysfunction is also associated with an increased expression of *KLF5* via FOXO1 and increased oxidative stress among cardiovascular patients with diabetes (Kyriazis et al., 2021).

Age‐associated marker *Nrf2* is essential to prevent oxidative stress; however, its lack is reported as a protection against the risk of atherosclerosis (reviewed by (Kloska et al., 2019)).

Furthermore, dysregulation among genes including *Nox4* (reviewed by (Chen et al., 2012)), *Cx43* (reviewed by (Michela et al., 2015)), *PGC1α* (reviewed by (Oka et al., 2020)), *VEGFA* (reviewed by (Braile et al., 2020)), and *TSP1* (reviewed by (Chistiakov et al., 2017)) are reported with increased risk of cardiovascular diseases. Another important gene associated with age‐related cardiovascular disease is *KL*, its genetic variant in Czech population is associated with a higher risk of cardiovascular diseases (Arking et al., 2005). The aging hallmark gene *SIRT1* is essential for the development of cardiomyocytes and protection against oxidative damage through an interplay with the circadian clock (Soni et al., 2021). In mouse hepatocytes and cultured fibroblasts, SIRT1 accumulation depicted circadian pattern, which was shown to be essential for the transcription of several core‐clock genes (*Bmal1*, *Per2*, *Cry1*) (Asher et al., 2008). Higher levels of *Sirt1* in mice is associated with cardiomyopathy (Alcendor et al., 2007), and alterations in circadian rhythms also increase the risk of cardiovascular diseases (El Jamal et al., 2023) and some of the genes described above are either regulated by or regulate elements of the circadian clock network. In mice liver, FOXO3 was reported to play a regulatory role in the transcription of *Clock* (Chaves et al., 2014). Cardiac ion channel *KChIP2* exhibit rhythmic expression in mice via *Klf15* and loss of *Klf15* resulted in an enhanced risk of arrythmias (Jeyaraj et al., 2012). Anea CB et al. reported the role of core‐clock machinery in regulating *Nox4* expression, and the loss of *Bmal1* in mice resulted in higher *Nox4* expression and oxidative stress in the aorta (Anea et al., 2013).

Altogether, these findings highlight the existence of altered clock‐associated aging mechanisms as additional risk factors for cardiovascular diseases (Figures 2 and 3).

#### 2.2.3 CNS aging‐related alterations

Circadian dysfunction is a common feature of aging‐related neurodegenerative diseases, including AD (Alzheimer's disease) (Leng et al., 2019; Musiek et al., 2015) and PD (Parkinson's disease) (Carter et al., 2021; Kondratova & Kondratov, 2012; Leng et al., 2020). Several resident cells of the CNS such as microglia and astrocytes showed an elevated expression of p16 and p21, which would result in senescence, thereby contributing to cognitive impairment and neurodegenerative diseases (Swenson et al., 2019). *PSEN1* and *PSEN2* genes, which are involved in the processing of amyloid precursor protein, have been linked to autosomal dominant early‐onset AD (Lanoiselee et al., 2017), and the *MAPT* gene, which codes for a protein involved in the stabilization of microtubules in neurons, has been associated with several neurodegenerative diseases, including AD, frontotemporal dementia, and PD (Allen et al., 2014; Strang et al., 2019).

Recent publications using both animal experimental models and human studies link circadian dysfunction to the pathological development of neurodegenerative diseases. Patients frequently experience disruptions in their sleep–wake cycles, melatonin secretion, and activity levels, which are known clock‐regulated physiological and behavioural outputs, and that decline with aging (Logan & McClung, 2019). Zeitgebers, such as bright light exposure, have been shown to improve motor and non‐motor symptoms among AD and PD patients (Fifel & Videnovic, 2018, 2019; Johnstone et al., 2015). One potential mechanism for circadian dysfunction in neurodegenerative diseases is the reported alteration in the expression of clock genes. In particular, the downregulation of clock genes was reported in mouse models for AD (*Per1*, *Per2*) (Wang et al., 2016), *BMAL1* in AD (Fan et al., 2022) and PD (Cai et al., 2010). As BMAL1 and CLOCK complex can interact with the dopamine pathway (Breen et al., 2014), this supports the involvement of circadian disruption in disease development. On the other hand, the accumulation of beta‐amyloid plaques in the brain, one key hallmark of AD, can interfere with the normal functioning of the SCN (Ahmad et al., 2023; Fifel & Videnovic, 2020). Variants of *APOE4* is associated with an increased risk of early onset AD (specifically homozygous risk allele e4), as well as genetic risk factor for late onset AD (presence of one or two copies of the e4 allele) (Cacace et al., 2016) and related dementias and sleep disturbances (Lim et al., 2013). In mouse cortex tissues derived from *Nes‐Bmal1 KO* (with intact central rhythms) and *a tamoxifen‐inducible global Bmal1* (*iKO*) mice (with disrupted central rhythms), and compared to the respective controls, elevated *Apoe* expression pointed to a role for fibrillar plaque formation regulated by the local *Bmal1* expression (Kress et al., 2018). Moreover, a study in a cohort of 296 AD patients (142 males and 154 females) and 423 controls (204 males and 219 female) reported an association between *APOE4‐*non‐carrier individuals and *BMAL1* (*rs2278749 T/C*) polymorphism (Chen et al., 2015). In PD, the loss of dopamine‐producing mesencephalic neurons in the brainstem (substantia nigra) may contribute to circadian dysfunction (Li et al., 2017). In addition, several cellular misfunctions including mitochondrial dysfunction, impaired protein degradation and aggregation, neuroinflammation, and cell death, impact the progression of PD and are all regulated by the clock (Kou et al., 2022; Sardon Puig et al., 2018). A recent study by our group reported that the knockout of the core‐clock genes *BMAL1* and *NR1D1* resulted in altered expression of *SNCA* (upregulation and downregulation, respectively), encoding for α‐synuclein considered as one of the hallmark proteins for the PD pathology (Yalçin et al., 2021). The knockout and knockdown of genes such as *SNCA* or *PINK1 are* implicated in the early‐onset phenotype of PD. In a h*SNCA* model of *Drosophila* (induced by TP‐αS or wt‐αS overexpression (Gajula Balija et al., 2011)) alteration in circadian behavioral outputs such as locomotor activity were observed and lengthening of the period was reported in older flies (De Lazzari et al., 2018). Certain genetic variants of clock genes, including *BMAL1* rs900147, *PER1* rs2253820 (Gu et al., 2015), *CLOCK* 3111T/C (Lou et al., 2018), and *Tef* rs738499 (Hua et al., 2012), have been linked to specific PD symptoms such as tremors, motor fluctuations, sleep disorders, and depression, respectively. Furthermore, weaker correlations in the expression of clock genes were reported in idiopathic PD patients compared to age and sex‐matched controls, suggesting the involvement of clock genes in distinct PD phenotypes, which exhibit daily fluctuations (Yalçin et al., 2021). A recent study by Pacelli et al. (2019) showed an interesting association between the clock and the *PARK2* gene, encoding for parkin, which is responsible for half of the autosomal recessive PD phenotype (Lunati et al., 2018). Normal fibroblasts showed rhythmic oscillations of glycolysis and mitochondrial activity whereas these rhythms were disrupted in patient‐derived fibroblasts, which were collected from early‐onset PD patients with carriers of *PARK2* mutation (Pacelli et al., 2019). The expression of core‐clock genes *CLOCK*, *PER3* and *NR1D1* showed a significant expression variation in patient‐derived fibroblasts compared to the controls. Therefore, understanding the mechanisms of circadian dysfunction in these aging‐associated pathologies may contribute for developing novel effective treatments and diagnostic strategies for these diseases based on the circadian clock status of the patients.

#### 2.2.4 Musculoskeletal system and aging

Aging results in a progressive decline in muscle mass and bone quality, thereby affecting mobility (Azzolino et al., 2021). Among different musculoskeletal diseases, sarcopenia (Dao et al., 2020) and osteoporosis (Aspray & Hill, 2019) present a major risk to aging society as they result in frailty and higher risk of falling (Cheng & Chang, 2017). Miller MJ et al. showed that *Atf4* is an essential mediator of skeletal muscle aging, and its loss in mice exhibited significant protection from age‐related decline in skeletal muscle strength (Miller et al., 2023). In a recent study from our group, we reported clock regulated expression of *Atf4* in human and mice skeletal muscle (Malhan et al., 2023). A cross‐sectional study performed on elderly women identified a specific polymorphism in *ACTN3* (R577X), which contributes to a higher risk of developing sarcopenia (Romero‐Blanco et al., 2021). Besides *ACTN3*, *NRF2* polymorphism is also associated with sarcopenia risks among elderly (Urzi et al., 2020). A systematic review reported several genes including *ACTN3*, *IGF1*, *TNFA*, *CNTF/R*, *UCP2/3* that showed association with skeletal muscle phenotype, and alterations with aging (Pratt et al., 2019). Interestingly, *IGF1* expression is clock regulated, and inhibition of IGF1‐signaling via caloric restriction promotes longevity among different species (reviewed by (Acosta‐Rodriguez et al., 2021)). Another risk factor for sarcopenia is over‐expression of *Dkk3*, its over‐expression in young mice leads to progressive muscle mass loss (Yin et al., 2018). In case of osteoporosis among elderly, genes like *CUL1*, *PTEN*, and *STAT1* depicted higher expression compared to non‐osteoporotic group (Deng et al., 2023). However, the interplay between *CUL1*, *PTEN*, *STAT1* and the circadian clock remains to be elucidated. Taken together, these findings point to a dysregulation of molecular pathways (IGF1, TNFA signaling) resulting in, at least, partial deterioration of musculoskeletal system with aging.

## 3 ALTERATION OF CIRCADIAN CLOCK AND SPACEFLIGHT: AN (ANTI)AGING EFFECT?

Space environment either promotes or even prevents aging processes. However, the exact duration of such an exposure to microgravity in Space among astronauts is still unclear, and when or to what extent pathophysiologic effects are actually detectable in an astronaut flown to Space remains a point of debate. Some of the mentioned dysfunctions seem to happen even faster in Space, a dramatic example is the reported loss of functional capacity of the cardiovascular system (e.g., cephalad fluid shift, heart rate, and stroke volume reduction), and musculoskeletal system (disuse atrophy), that proceeds approximately 10 times faster in Space than due to normal aging on Earth (Vernikos & Schneider, 2010). For example, a study using rat neuronal cells (PC12 cells) under simulated microgravity condition showed partial cell cycle G1 phase arrest, and activation of p53 and p16 pathways, that leads to cellular senescence and aging in vitro (Wang et al., 2009). In tail‐suspended mice (analogue to microgravity unloading), mimicking spaceflight condition, a decreased lymphocyte B‐cell differentiation was observed resembling aging‐related modifications (Lescale et al., 2015).

A number of bedrest studies with otherwise healthy participants (analogue to human spaceflight) have been used to simulate physiological alterations related to acute or chronic muscle disuse, similar to microgravity environments as experienced during short‐, medium‐, and long‐duration spaceflights, were reported to lead to aging‐related phenotypes, such as deterioration of cardiovascular function and affecting musculoskeletal health (reviewed by (Kehler et al., 2019)).

Several studies both on experimental animal models and astronauts examined the impact of the Space environment on aging and age‐associated changes (Biolo et al., 2003; Fukuda et al., 2021; Garrett‐Bakelman et al., 2019; Honda et al., 2012; Le Bourg, 1999; Lescale et al., 2015; Ma et al., 2015; Nwanaji‐Enwerem et al., 2020; Otsuka et al., 2022; 2019; Strollo, 2000; Takahashi et al., 2021; Vernikos & Schneider, 2010; Wang, 1999; Wang et al., 2009). During spaceflight, astronauts experience numerous disruptions of physiological functions including muscle atrophy (Fitts et al., 2010), bone loss (Narici & de Boer, 2011), sleep disruption, and behavioural changes (Barger et al., 2014), which, to some extent, resemble alterations observed during aging on Earth. Physiological function, as well as behavior, is also regulated by the circadian system and a circadian clock malfunction can lead to several pathological conditions as described in the previous sections (e.g., sleep disorders, cardiovascular impairments) (Ma et al., 2015).

### 3.1 Effects of microgravity on organ systems

As circadian rhythms on Earth are governed by natural diurnal light–dark cycles, it will be of utmost importance to better understand the role of the circadian system and its influence on body function for health and performance of astronauts under extreme environmental conditions of artificial light–dark cycles in their future spaceships and planetary habitats (Moon, Mars) on long‐duration missions. In microgravity, the shift of fluids from the lower body toward the head and heart (cephalad fluid shift) causes a lower blood production and abnormal blood pressure resulting in a decreased stroke volume and diastolic blood pressure with a lower heart rate (Norsk et al., 2015; Otsuka et al., 2015). With less volume to pump, the heart muscle adapts accordingly with size reduction of the left ventricle wall and lower contractility (Perhonen et al., 2001). Due to microgravity‐induced functional adaptation of the cardiovascular system after the first 1 or 2 weeks in Space, though more or less suffering from acute “space‐sickness” during initial microgravity adaptation, the astronaut may be able to still maintain performance and mission duties during their entire missions (Goswami, 2017). However, when re‐entering gravitational forces, astronauts suffer from orthostatic intolerance with risk of syncope shortly after landing (Goswami, 2017). While muscle atrophy, bone loss and blood volume may be partly prevented by strict exercise as an inflight countermeasure (Loehr et al., 2015) and thus may allow for improved mission success following longer stays in Space, other impairments such as SANS create an even greater problem for extended Space missions over several weeks or months. SANS includes optic disc edema, posterior globe flattening, chorioretinal folds, and retinal nerve fiber layer thickening (Ong et al., 2022). Pathogenesis of SANS is still unknown, but underlying hypothesis include an elevated intracranial pressure based on the known cephalad fluid shift with an upward displacement of the brain (Van Ombergen et al., 2017), and activated inflammatory or oxidative stress pathways (Ong et al., 2022).

Another space‐related challenge for astronauts is sleep disruption. Astronauts experience sleep fragmentation (i.e., impaired sleep architecture), reduced daytime alertness, and reduced nocturnal sleep tendency (Czeisler et al., 1991).

### 3.2 Clock genes and gravitational unloading in animal and human research

Microgravity‐induced biological dysregulations discussed in the previous section may be explained by disruptions in the circadian clock (Czeisler et al., 1991). During spaceflight many environmental factors that have been reported as possible zeitgebers are altered (e.g., 24 h light–dark cycle, and feeding habits). The expression of clock‐associated genes may be changed by many other factors related to the microgravity environment in Space (e.g., electromagnetic field, radiation, hypoactivity, and heat stress) via lack of sweat evaporation required for body core temperature control, (see (Stahn et al., 2017)), as well as unique conditions onboard the ISS (International Space Station) (e.g., lighting conditions, shift‐work schedule) (Guo et al., 2014). Although the effect of spaceflight on the circadian rhythm is known for more than 30 years, little progress has been made on the exact mechanism of circadian dysregulation at the molecular level. Even though, the effect of spaceflight on the human circadian clock has not been investigated in greater detail, studies on space flown rodents and *Drosophila* suggested a key role for several clock genes in microgravity‐associated pathological alterations. Ma et al. reported that in 13 days space flown *Drosophila* the locomotor activity rhythm and sleep pattern remained normal and the expression of some major clock genes (*Per*, *Tim*, *Vri*, *Cry*) were unaffected (Ma et al., 2015). Regulated output genes of the circadian clock system, CCGs, differed between space flown flies and ground control group suggesting an affected output pathway. Genes such as *Ilp3*, *Act87E* (cytoskeleton), *Mlp60A* (zinc ion binding), *Tutl* (synaptic target recognition) were upregulated and lipoprotein genes (*Kif3C*, *Nfl*) were downregulated. However, the authors noted that a 48 h delay in sampling could have affected the results (Ma et al., 2015). Ogneva et al. reported on multiple generations of *Drosophila* flown in Space for 44.5 days. Comparison between generations and ground control revealed differentially regulated biological processes and altered molecular functions including cytoskeleton protein binding, protein kinase activity, electron carrier activity, heme binding, and structural constituents of the cuticle (Ogneva et al., 2016). In 2014, the HEART flies mission launched, and a system‐specific transcriptomic analysis on the fly hearts was performed upon return (Walls et al., 2020). Overall, the study showed a large number of changed genes in microgravity conditions in mutant and WT flies. Microgravity resulted in downregulation of collagen network remodelling and degradation genes such as *MMP1*, multiple collagen‐type‐IV‐encoding genes (*Cg25C*, *Viking*, *Col4a1*, *Prc*), and upregulation of proteolysis genes, carbohydrate metabolic process genes (Walls et al., 2020). In conclusion, the results suggested that alterations in proteostasis (Figure 1; right panel) contribute to space‐associated cardiac dysfunction (Walls et al., 2020).

So far, circadian clock genes have not been investigated thoroughly in humans during spaceflight, but animal studies and in vitro studies have been performed and shed some light on the topic. Kumar et al. reported that in 15 days space flown mice a persistent increase in oxidative stress‐related target genes can be seen. Regulatory genes such as *Nrf2* and *Tnf* expression are downregulated in Space though against expectations an upstream key modulator *Tp53* remained unchanged (Kumar et al., 2021). An increase of oxidative stress can be linked to an increase in ROS that occur in microgravity due to radiation and altered gravity (Takahashi et al., 2017). ROS accumulation causes cell death, cell senescence, and cell repair failure and induces cellular pathways of inflammation (Gómez et al., 2021). Muid et al. found an elevated amount of IL6, a pro‐inflammatory modulator, in HUVEC (human umbilical vein endothelial cells) on board the ISS after 3 months (Muid et al., 2013). In another study, Washington et al. found elevated levels of *IL6* expression in wild type mice after hindlimb suspension in comparison to *IL6* knockout mice suggesting that IL6 has a key role in muscle growth after disuse atrophy (Washington et al., 2011). In a study with *Igf1* knockout mice, the authors reported an equal muscle mass loss after hindlimb suspension between knockout mice, their controls (knockout without hindlimb suspension) and wild type (no knockout with hindlimb suspension). The loss of muscle strength was greatest in *Igf1*‐deleted mice following disuse. The return of muscle mass and strength in recovery seemed independent of *Igf1* concluding *Igf1* as one of the key players in muscle degradation (Spradlin et al., 2021). Vogel et al. reports on human cells (Jurkat T‐cells, U937) that were exposed to altered gravity within short duration gravitational transitions in parabolic flight (Vogel et al., 2019). The expression of *HIF1A*, a regulator of T‐cell response, was downregulated in microgravity, as well as pro‐inflammatory cytokine *IL1b*. *HIF1*‐related genes (involved in HIF1 signaling) were upregulated during the hypergravity phase, but not reversed or otherwise changed during microgravity phase (Vogel et al., 2019). A previous study of the same working group discovered altered regulatory genes in microgravity phase (*ATP6V1*, *LIN00837*, *IGHD3*) (Thiel et al., 2017).

To obtain a comprehensive view of human body's response to Space environment, the NASA carried out a twin's study in 2015–2016 where one identical twin astronaut was monitored before, during, and after a one‐year mission while the other twin counterpart served as a genetically matched ground control (Garrett‐Bakelman et al., 2019). The results from the twin's study revealed multiple changes including, increased retinal thickness, and changes in artery dimensions due to Space environment, that reversed to their normal levels after 6 months upon return to Earth. Interestingly, telomere lengthening (sign of anti‐aging) was observed while the astronaut was in Space, which reversed upon his return to Earth. Following 6 months after return to Earth, the twin also showed increased number of shorter telomeres (sign of aging). Moreover, increased DNA damage, and altered cognitive function were observed after 6 months on Earth (Garrett‐Bakelman et al., 2019). An interesting anti‐aging effect of spaceflight was reported in the DNA methylation data of astronauts on Space mission for 520 days (Nwanaji‐Enwerem et al., 2020). In another study, space flown *Caenorhabditis elegans* exhibited the signs of slower aging through neuronal and endocrine response (Honda et al., 2012). Similarly, *Drosophila melanogaster* post 13 days in Space showed longer lifespan versus their ground controls (Ma et al., 2015). Furthermore, Otsuka K et al. reported anti‐aging effect of spaceflight through evaluation of heart rate variability among astronauts during 6‐month mission (Otsuka et al., 2019), and during 1‐year mission (Otsuka et al., 2022). In vitro culture of human skeletal muscle myoblasts (which can fusion to multinucleated myotubes/myofibers and eventually differentiate into young and adult muscle fibers) under simulated microgravity resulted in decreased cell proliferation and cytoskeleton enlargement of cells similar to changes observed during normal aging (Takahashi et al., 2021). Also in vitro experiments in Yeast fungi, *Saccharomyces cerevisiae*, seem to point to a correlation between aging‐induced phenotype due to altered gravity conditions, and signs of accelerated aging including oxidative stress and heat shock protein expression under simulated microgravity (Fukuda et al., 2021).

### 3.3 Outlook to future research

Hence, the results presented in space‐analogue studies, space flown mice or isolated cells housed in specialized animal habitats or single‐cell culture modules, have shown that microgravity alters circadian clock genes in various organisms from cells (Table 2), small animals to humans. Several circadian clock genes are major regulatory genes affecting downstream pathways resulting in upregulation of pro‐inflammatory pathways, and downregulation of anti‐oxidative pathways. Inflammation and oxidative stress are suspected to cause physiological function impairments such as SANS. Space travel evolves toward commercialized flights and longer duration missions to Moon and Mars. Both require thorough preparation regarding already known (e.g., radiation, microgravity), and possible yet unknown health risk factors, for example, prolonged isolation, psychology factors such as crew teaming‐up issues or living on spacecrafts and future planetary habitats in distances far away from home on Earth. Thus, investigating the circadian clock and its misalignments in Space is of major relevance to get a deeper understanding of the potential key roles in many functional impairments in human physiology on the ground and in spaceflight. A recent comprehensive bioinformatics and computational analysis using 1179 mammalian skeletal muscle samples from 28 published genomics and proteomics datasets, by our group showed common gene expression changes in the circadian clock and skeletal muscle associated pathways due to aging on Earth and long‐term spaceflight in mammalian skeletal muscle (Malhan et al., 2023).

**TABLE 2:** Aging‐related studies that were/are carried out by international space agencies (NASA, ESA, CSA [Canadian Space Agency], and JAXA; d = day[s]).

	Study description	Species	Duration in space	Main findings	References
NASA	Differential effects on telomeres and telomerase in twin astronauts associated with spaceflight (03/2015–03/2016)	Human	Inc 43/44 Inc 45/46	Identification of pathways and mechanisms that may be vulnerable to spaceflight; majority of the human health can be sustained over a 340‐day Space mission	Garrett‐Bakelman et al. (2019) Luxton and Bailey (2021) Bailey et al. (2022) Luxton, McKenna, Lewis, et al. (2020) Luxton, McKenna, Taylor, et al. (2020)
ESA	Skin‐B Improving understanding of skin aging (03/2013–04/2017)	Human	Inc 35/36 Inc 37/38 Inc 39/40 Inc 41/42 Inc 43/44 Inc 45/46 Inc 47/48 Inc 49/50	Skin symptoms recorded onboard ISS mainly correlated with poor hygiene; spaceflight under current conditions has no negative impact on skin physiological parameters	König et al. (2015) Braun, Thomas, et al. (2019) Braun, Binder, et al. (2019) Theek et al. (2020)
NASA	Impact of space radiation on cognition. Synapses and biomarkers in aging and Alzheimer's disease (06/2014–05/2018)	Mouse	—	Low‐dose ^56^Fe irradiation generally increased inflammation; physiological and corresponding behavior effects may relate to sex and genetic predispositions	Schroeder et al. (2021) Liu et al. (2019)
NASA	Effects of weightlessness on the embryonic development and aging of *Drosophila* Aim: Behavior response, sexual behavior, and aging of drosophila in weightlessness (1975 (Cosmos 782))	*Drosophila*	20 d	The development of *Drosophila* was insensitive to weightlessness and the aging processes were not influenced, except for a slight reduction in the amount of lipofuscin present in the midgut and Malpighian tubules.	Miquel and Souza (1991) Miquel et al. (1974) Miquel (1982)
NASA	Aging and cardiopulmonary function Aim: Longitudinal study of astronauts cardiopulmonary function (1989–2010)	Human		Overall mortality is higher in astronauts probably due to higher risk of spacecraft accidents; astronauts have a higher risk of developing cataract as they are exposed to a higher dose of space radiation.	Institute of Medicine Committee on the Longitudinal Study of Astronaut (2004)
CSA	The Space environment causes acceleration of vascular aging: roles of hypogravity, nutrition and radiation Aim: Provide insights into the mechanism of cardiovascular aging and diabetes development (2019–2022)	Human	Exp. 65 (184 d) Exp. 66 (164 d) Exp. 61 (126 d) Exp. 60 (101 d) Exp. 63 (187 d) Exp. 64 (177 d)	‐	Analysis ongoing
NASA	Association between aging and specific physiological measurements Aim: Longitudinal study of astronaut health (1989–2010)	Human		Among general population, LDL cholesterol level increased and HDL level decreased with age. While, in astronauts, HDL level showed no dramatic change with age.	Institute of Medicine Committee on the Longitudinal Study of Astronaut (2004)
NASA	Rodent research‐17 Hypothesis: Exposure to microgravity accelerates changes observed during aging process (2019)	Mouse	SpaceX_18 (32 d, 22 h)	‐	Analysis ongoing
NASA	Effects of weightlessness on the genetic and aging process of *Drosophila* (1977 (Cosmos 936))	*Drosophila*	19 d	The reduced vitality and the short life span manifested by the flies which were exposed to hypogravity during the first days of their imaginal life suggests that aging process may be accelerated during spaceflight	Miquel and Philpott (1978) Miquel and Souza (1991) Miquel et al. (1975) Miquel (1982) Philpott et al. (1974)
NASA	Vision and aging in the astronaut population Hypothesis: Prevalence of eye diseases in the astronaut population (1989–2010)	Human	—	—	No info available
NASA	Rodent Research 8 Investigate the physiology of aging and the effect of age on disease progression (2018–2019)	Mouse	SpaceX_16 (39 d)	—	Analysis ongoing
NASA	Possible Effects of Zero Gravity on Radiation‐induced somatic damage (1967)	*Drosophila*	Biosatellite II (2 d)	Elimination of the effect of 2500 R by spaceflight in Habrobracon oogonia where the effects of 500 R normally can be easily observed	von Borstel et al. (1969)
JAXA	Mechanism of accelerated aging under microgravity Aim: measuring biomarkers for mineral metabolism and determine their role in accelerated aging of astronauts (2020–2022)	Human	Exp. 65 (184 d) Exp. 66 (164 d) Exp. 63 (187 d) Exp. 64 (177 d)	—	Analysis ongoing
NASA	Aging and blood pressure in astronauts Longitudinal study to examine blood pressure in astronauts (1989–2010)	Human	—	Overall the comparison group showed higher blood pressure than the astronauts.	No info available
ESA	Effects of prolonged spaceflight on DNA methylation age (2020–2022)	Human	Exp. 65 (184 d) Exp. 66 (164 d) Exp. 63 (187 d) Exp. 64 (177 d)	—	Data collection ongoing (09/2023)
ESA	Blood and oxidative stress Aim: Cell aging due to oxidative stress (2004–2005)	Human	Exp. 10 (193 d) Exp. 11 (179 d)	Single‐case study indicates that erythrocytes decrease their antioxidant defences to counteract oxidative stress during short spaceflight	Rizzo et al. (2007)
CSA	Cardiovascular health consequences of long‐duration spaceflight (2009–2013)	Human	Exp. 23 (75 d) Exp. 29 (40 d) Exp. 35 (58 d) Exp. 21 (51 d) Exp. 22 (109 d) Exp. 24 (117 d) Exp. 26 (111 d) Exp. 30 (166 d) Exp. 37 (61 d) Exp. 34 (117 d)	Astronauts suffer from orthostatic intolerance after spaceflight; cardiovascular deconditioning may occur despite countermeasure exercise	—
NASA	Pulmonary Function and Aging in LSAH Participants Aim: Pulmonary function in astronauts (1989–2010)	Human	—	—	No info available
NASA	Association between age and PSA values in LSAH participants (1989–2010)	Human	—	Establishment of appropriate reference ranges for free, complexed and total PSA (prostate specific antigen)	Oesterling et al. (1995)
NASA	Systemic therapy of NELL‐1 for spaceflight induced osteoporosis Aim: to study accelerated bone loss in Space similar to aging and try new therapeutics (2017)	Mouse	SpaceX_11 (30 d)	—	Analysis ongoing
NASA	Human sleep, circadian rhythms and performance in Space (1996)	Human	17 d	No evidence that 17 d spaceflight disrupts human circadian time‐keeping system, but sleep was disrupted.	Monk et al. (1998) Monk et al. (1999) Monk (1999)
NASA	Rodent Research 3 Aim: Determine if inhibition of myostatin prevents bone loss Assess if preventing muscle loss mitigates bone loss Quantify effects of spaceflight on molecular markers of muscle atrophy and bone loss (2016)	Mouse	SpaceX_8 (33 d)	Identification of tissue‐independent biological pathways that are dysregulated due to space radiation dose	McDonald et al. (2020)
ESA	Cardiovascular adaptation to weightlessness (2003–2004)	Human	Exp. 7 (185 d) Exp. 8 (195 d)	Neural mechanisms of heart rate regulation recover needs 1 month to recover after long duration mission	Vandeput et al. (2013)
JAXA	Phospho‐aging mechanism of accelerated aging under microgravity Hypothesis: Mechanism of microgravity induced aging is identical to phosphate induced aging (2020–2022)	Human	Exp. 63 Exp. 65 Exp. 67 Exp. 64 Exp. 66	—	Analysis ongoing

Future spaceflight experiments can explore clock‐related gene expression changes in human tissue samples, for example, derived from liquid biopsies (e.g., blood, saliva, urine) as soluble gene transcripts and proteins (body fluids and/or systemic approach) (Dose et al., 2023). Alternatively, they can be studied in an expedient way using small biopsy samples obtained directly from a tissue of interest (tissue‐specific approach) such as in a particular postural and gravity‐sensitive functional leg skeletal muscle as recently reported from Space omics datasets of astronauts (Blottner et al., 2023; Rittweger et al., 2018).

New findings from this still largely unstudied area will help to improve countermeasures for future Space missions, and at the same time increase our understanding toward life and aging on Earth.

## 4 CONCLUSIONS

Aging is a complex process that is influenced by a multitude of factors, including circadian regulation. The disruption of circadian rhythms can have profound consequences for human health, particularly during spaceflight where astronauts are exposed to extreme conditions that can disrupt their normal daily mission duties including impaired sleep–wake cycles. Furthermore, aging on Earth is also associated with a decline in circadian regulation, which can contribute to the development of various age‐related diseases such as visual decline, cardiovascular diseases, neurodegeneration, and musculoskeletal deterioration.

Understanding the mechanisms underlying circadian regulation in aging is thus essential for developing interventions that can improve health outcomes for both astronauts and elderly population on Earth. For instance, promoting healthy sleep patterns through the use of light therapy or other non‐pharmacological interventions may help to mitigate the negative effects of circadian disruption during spaceflight. Melatonin supplementation may help to minimize the impact of oxidative stress seen due to aging or due to extreme environmental conditions. Interventions that target circadian regulation in aging individuals on Earth may help to reduce the risk of age‐related diseases and improve overall health and well‐being.

Moving forward, it will be important to continue to explore the relationship between aging, circadian regulation, and spaceflight. Advances in technology and data analytics are providing new opportunities to monitor and manipulate circadian rhythms in real‐time, which could have important implications for human health in Space and on Earth. Additionally, the development of personalized interventions that specifically target circadian rhythms may hold promise for improving health outcomes among elderly. By better understanding the complex interplay between these factors, from single cells and tissues to multicellular organs and systems level, but also from various organisms in evolution up to higher vertebrates and humans, we may be able to develop new strategies for improving health outcomes and promoting healthy aging for all individuals.

## AUTHOR CONTRIBUTIONS

Angela Relόgio involved in conceptualization, funding acquisition, investigation, supervision, writing, reviewing, and editing. Deeksha Malhan involved in conceptualization, investigation, visualization, writing, reviewing, and editing. Britt Schoenrock involved in investigation, writing, reviewing, and editing. Müge Yalçin involved in investigation, writing, reviewing, and editing. Dieter Blottner involved in funding acquisition, investigation, supervision, writing, reviewing, and editing. All authors have read and agreed to the final version of the manuscript.

## FUNDING INFORMATION

The work in the group of A.R. was funded by the Dr. Rolf M. Schwiete Stiftung. M.Y. was additionally funded by the Berlin School of Integrative Oncology (BSIO) graduate program of the Charité Medical University Berlin. D.B. and B.S. were supported by a grant from the Federal Department of Economy and Climate protection (BMWK) through Deutsches Zentrum für Luft‐ und Raumfahrt (DLR e.V., Bonn‐Oberkassel, Germany, #50WB2029 to D.B.).

## CONFLICT OF INTEREST STATEMENT

The authors have no conflict of interest to declare.

## Data Availability

Data sharing is not applicable to this article as no new data were created or analyzed in this study.
